# A prognostic signature associated with cell senescence predicts survival outcomes and strongly associates with immunotherapy and chemotherapy response in breast cancer

**DOI:** 10.1097/MD.0000000000034018

**Published:** 2023-06-16

**Authors:** Tao Pan, Zhengfang Hu, Dongyan Xu, Yunxiang Zhou, Suzhan Zhang, Yiding Chen

**Affiliations:** a Department of Breast Surgery, The Second Affiliated Hospital, Zhejiang University School of Medicine, Hangzhou, China; b Beijing Tian Tan Hospital, Capital Medical University, Beijing, China; c Key Laboratory of Cancer Prevention and Intervention, Ministry of Education, The Second Affiliated Hospital, Zhejiang University School of Medicine, Hangzhou, China.

**Keywords:** breast cancer, consensus clustering, immune infiltration, nomogram, senescence

## Abstract

The objective of this study is to assess the predictive potency of cell senescence-related genes (CSRGs) in breast cancer (BC) and establish a risk signature. Trascriptome data of CSRGs were obtained from the TCGA and GEO databases. Consensus clustering was used to generate CSRGs-based molecular clusters for BC patients. A CSRGs-derived risk signature was built using multiple Cox regression analyses of differentially expressed genes (DEGs) between clusters. The prognosis, immune infiltration, chemotherapy and immunotherapy response between different risk groups were analyzed and compared. Two molecular clusters of BC patients were generated on the basis of 79 differentially expressed CSRGs, which showed distinct prognosis and immune infiltration. A total of 1403 DEGs between the CSRGs-derived clusters were found, and 10 of them were independent prognostic genes that used to construct a risk signature. The results demonstrated that patients with older age and advanced stage presented with a higher risk scores. In addition, the risk signature was found to be associated with outcomes, immune infiltration, chemotherapy and immunotherapy response. Patients in the low-risk group showed a favorable prognosis and higher immunotherapy response than those in the high-risk group. Finally, we developed a highly stable nomogram that incorporates risk signature, chemotherapy, radiotherapy, and stage variables, enabling accurate prediction of the overall survival (OS) of individual patients. To conclude, the signature derived from CSRGs holds great promise as a biomarker for prognostic assessment of BC and may serve as a valuable tool in guiding immunotherapy.

## 1. Introduction

Breast cancer (BC) is the most prevalent cancer among women worldwide, characterized by high morbidity and mortality rates.^[1]^ In the United States, approximately 276,480 new cases of invasive BC and 42,170 deaths among women are predicted to occur in 2020.^[2]^ Despite substantial progress in treatment modalities, the mortality rate for BC continues to be unacceptably high.^[3]^ The immune microenvironment plays a critical role in the pathogenesis and treatment of cancer, particularly through the infiltration of diverse immune cell populations including T cells, B cells, and NK cells. The quantity and phenotype of infiltrating immune cells have been shown to influence tumor growth, progression, and response to immunotherapy. Despite recent advances in our understanding of the immune microenvironment, the precise molecular mechanisms that underlie these effects remain poorly characterized.^[4]^ Therefore, it is imperative to unravel the molecular mechanisms underlying BC pathogenesis to advance cancer therapeutics.

Cellular senescence is a biological response to various intrinsic and extrinsic stimuli aimed at eliminating senescent cells and preserving cellular homeostasis.^[5]^ This process can be triggered by multiple factors, including oxidative stress, genotoxic stress, telomere structural changes, etc.^[6]^ The accumulation of cellular damage may result in either cell senescence or cancer. The relationship between cellular senescence and cancer is complex and multifaceted, as emerging evidence suggests that senescence may exert both pro-tumorigenic and anti-tumorigenic effects. Senescence can induce a permanent cell growth arrest, thereby preventing tumor formation. However, the accumulation of senescent cells due to inefficient clearance mechanisms can lead to the promotion of tumorigenesis and progression.^[7]^

Several prediction models derived from cell senescence-related genes (CSRGs) have been developed to predict prognosis and treatment efficacy for multiple types of cancer, highlighting the critical role of these genes in cancer biology.^[8,9]^ In this study, we firstly generated molecular clusters derived from CSRGs, and then established a risk signature for the overall survival (OS) prediction of BC patients. Differences in clinical characteristics, immune infiltration, chemotherapy and radiotherapy response between different risk groups were further analyzed.

## 2. Materials and methods

### 2.1. Data collection

We obtained RNA-seq and clinical data from BC patients in the TCGA database (TCGA-BRCA cohort), which served as the training set to develop a molecular clusters based on CSRGs. We further retrieved and prepared a validation cohort from the GEO database (GSE96058 cohort), which consists of 3409 BC patients. In total, 432 CSRGs were obtained from the in the Molecular Signatures Database genetic database.

### 2.2. Consensus clustering

Differentially expressed CSRGs between tumor and normal tissues was identified using the Limma package, and the resulting differentially expressed CSRGs were subjected to consensus clustering analysis using the Consensus Cluster Plus package. To determine the ideal number of clusters, we employed the consensus matrix and cumulative distribution function in our analysis. These statistical methods offer valuable insights into the structure and stability of clustering, facilitating the identification of robust and biologically relevant cluster solutions. To identify clusters that were most relevant to survival, the Kaplan–Meier method was employed.

### 2.3. Development and validation of a risk signature

Univariate Cox regression analysis was performed to select the prognostic differentially expressed genes (DEGs) between CSRGs-derived clusters. To avoid overfitting, LASSO regression analysis was executed to select signature genes via the glmnet package. Independent prognostic genes were identified using multivariate Cox regression analysis. The risk score was computed using the following formula: riskscore = $\underset{n}{\sum^{n}}Coef\left(i\right)*Expr\left(i\right)$. BC patients were stratified based on the median risk score into low and high-risk cohorts. The prognostic value of these risk cohorts was assessed utilizing the Kaplan–Meier survival analysis. Furthermore, to validate the predictive accuracy and stability of the risk score model, we constructed a receiver operating characteristic curve.

### 2.4. Immune cell infiltration and immunotherapy response

To investigate immune cell infiltration in the TCGA-TGCT cohort, the CIBERSORT package was applied to compare 22 immune cell types between different risk groups and CS-derived clusters. Transcriptome data of 24 immune check inhibitor genes were extracted and compared between risk groups. Furthermore, we leveraged the Cancer Immunome Atlas to assess the efficacy of immunotherapy, wherein we computed the immunophenoscore (IPS) for each unique sample. The Tumor Immune Dysfunction and Exclusion (TIDE) algorithm was utilized to anticipate the clinical response to immune checkpoint inhibitors. The parameters used for comparison included TIDE, CD274, cancer-associated fibroblast, Merck18, interferon-gamma, and myeloid-derived suppressor cells.

### 2.5. Functional enrichment analysis

In order to unravel the putative biological pathways associated with the low- and high-risk cohorts, functional enrichment analyses were performed using Gene Ontology (GO) and Kyoto Encyclopedia of Genes and Genomes (KEGG) databases on the DEGs between the CSRGs-derived clusters, using the clusterprofiler software package.

### 2.6. Evaluation of the drug sensitivity

To determine the association between drug sensitivity and senescence-associated signatures, the “pRRophetic” R package was employed to derive the half-maximal inhibitory concentrations (IC50) of drugs. The IC50 values of low- and high-risk cohorts were subjected to Wilcoxon signed-rank tests for comparison.

### 2.7. Nomogram construction

To identify the independent risk factors for BC, we utilized both univariate and multivariate Cox regression analyses. Using the rms package, a nomogram was constructed by integrating all independent risk factors. The predictive performance of the established nomogram was evaluated using C-index and calibration plots. Decision curve analysis was conducted using the rmda package to assess the benefit of all strategies used for OS prediction.

### 2.8. Statistical analysis

The R software (version 4.2.0) was utilized for data analysis and to generate the relevant figures. The risk scores for different clinical feature groups were compared using either Wilcox.test or anova. Kaplan–Meier survival analyses were executed, and log-rank tests were performed to assess the disparities in OS among the target groups. A statistical significance level of *P* < .05 was deemed as the threshold.

## 3. Results

### 3.1. Senescence-related clusters in BC patients

**Figure 1.**
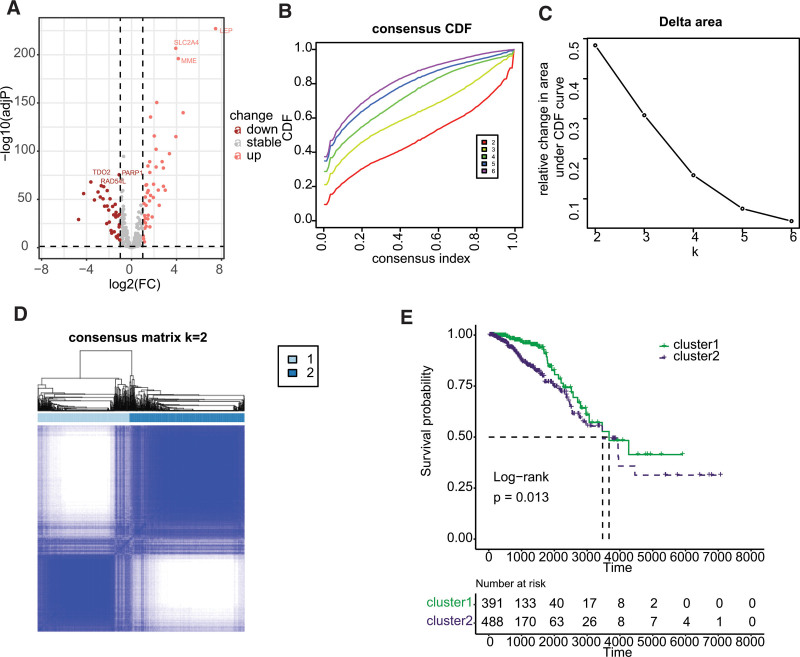
Consensus clustering of BC based on differently expressed cellular senescence-related genes. (A) Differential expression analysis of cellular senescence-related genes. (B) Consensus CDF in consistent clustering (k = 2–9). (C) Relative change in area under the CDF curve from k 2 to 9. (D) Consensus heatmap defining the 3 clusters (k = 3). (E) Survival analysis of BC subtypes derived from cell senescence-related genes. BC = breast cancer, CDF = cumulative distribution function.

**Figure 2.**
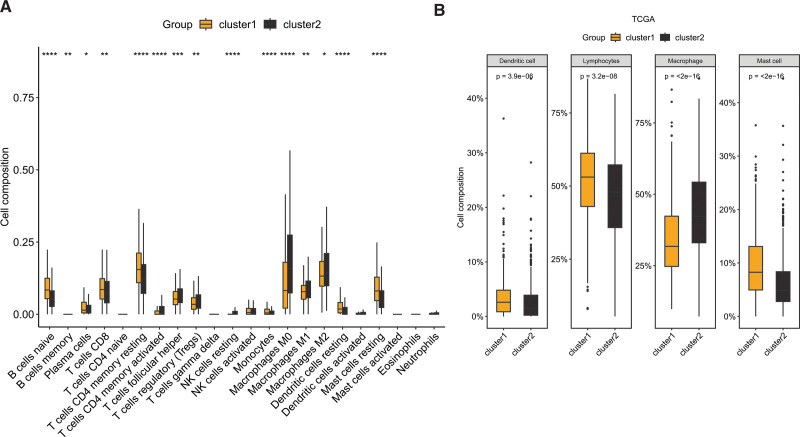
Immune infiltration analysis among BC subtypes derived from cell senescence-related genes. (A) Infiltration analysis of 22 immune cells based on the CIBERSORT algorithm. (B) Comparison results after classifying the 22 immune cells into 4 categories. *P < .05, **P < .01, ***P < .001, ****P < .0001. BC = breast cancer.

Differential expression analysis revealed that 79 cell-related genes exhibited aberrant expression in BC, with 44 genes showing significant upregulation and 35 genes displaying significant downregulation, as illustrated in Figure 1A. Consensus clustering analysis was then performed using these differentially expressed cellular senescence-related genes, and the results demonstrated a significant difference when k = 3 for the flat slope curve (Fig. 1B–D). Consequently, the TCGA-BRCA cohort was divided into 2 clusters, namely cluster1 and cluster2. K-M survival analysis revealed that cluster1 had a better prognosis than cluster2 (Fig. 1E, *P* = .013). Analysis of immune cell infiltration indicated significant differences between cluster1 and cluster2, with naive B cell, memory B cell, plasma cell, CD8 T cell, resting CD4 memory T cell, monocyte, resting dendritic cell (DCs), and resting mast cell showing enrichment in cluster1, while activated CD4 T cell, follicular helper T cell, regulatory T cell, resting NK cell, and macrophages exhibited higher infiltration in cluster2 (Fig. 2A). Upon classifying the immune cells, we reanalyzed the data and discovered that dendritic cell, lymphocytes, and mast cell were significantly enriched in cluster1, whereas macrophage was significantly enriched in cluster2 (Fig. 2B).

### 3.2. Establishment of senescence-related signature

**Figure 3.**
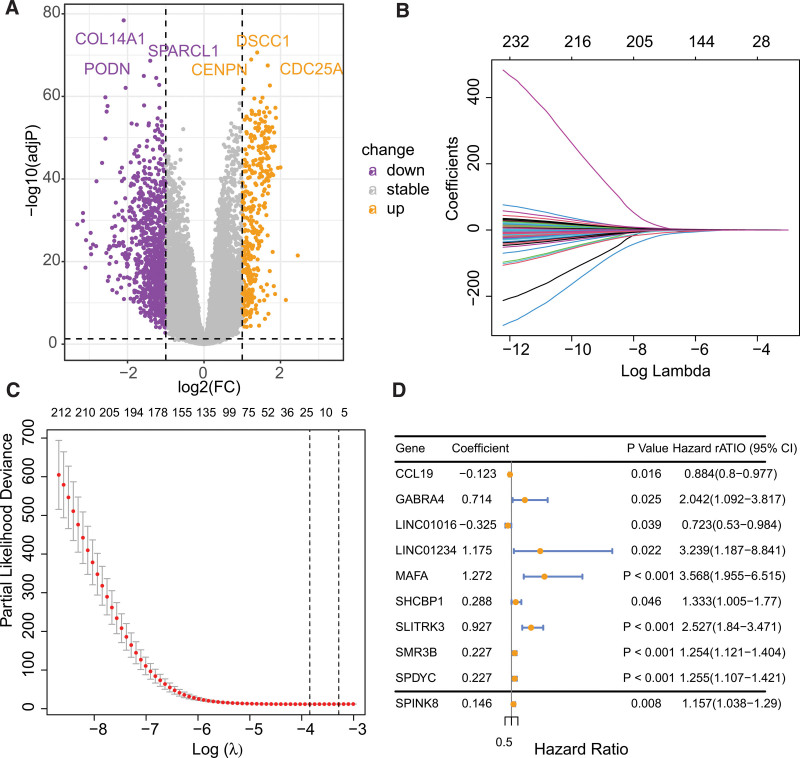
Construction of the cell senescence-related gene-based BC cell risk feature. (A) Differential expression gene analysis among BC subtypes derived from cell senescence-related genes. (B) Coefficients of the LASSO analysis. (C) The senescence-related signature obtained 6 prognostic genes with a minimum lambda value. (D) The forest plot of hazard ratios for the final 9 genes included. BC = breast cancer.

**Figure 4.**
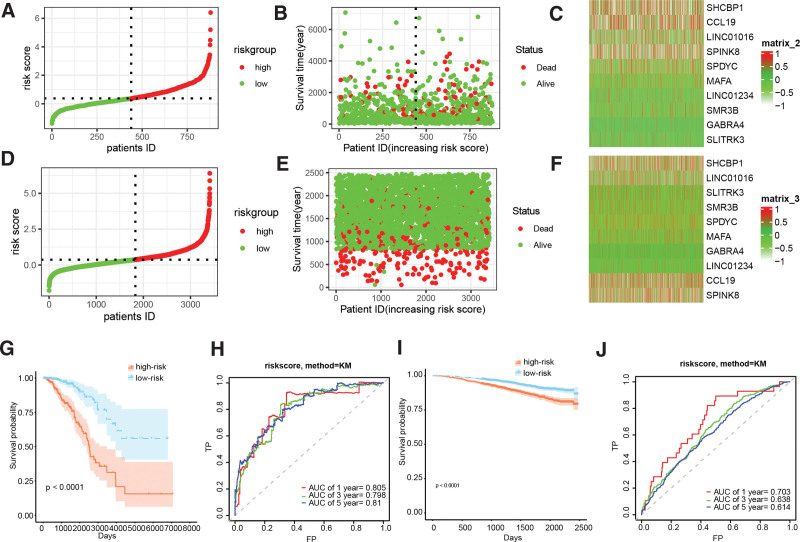
Performance evaluation of the BC prognostic model derived from cell senescence-related genes. (A and D) Survival curve of BC patients across TCGA-BRCA and GSE96058 cohorts. (B and E) Survival status of BC patients across TCGA-BRCA and GSE96058 cohorts. (C and F) Heatmaps displaying the expression levels of signature genes in THCA patients across the TCGA-BRCA and GSE96058 cohorts. (G and I) Survival analyses of different risk groups in the TCGA-BRCA and GSE96058 cohorts. (H and J) ROC analyses of the risk model in the TCGA-BRCA and GSE96058 cohorts. BC = breast cancer, ROC = receiver operating characteristic.

Differential gene expression analysis revealed 1403 genes that were differentially expressed between cluster1 and cluster2, of which 1026 genes were downregulated and 377 genes were upregulated (Fig. 3A). Univariate Cox regression analysis indicated that out of these genes, 235 genes displayed a significant correlation with the prognosis of BC. Subsequently, LASSO analysis was performed to narrow down the candidate genes and identified 18 prognostic genes (Fig. 3B and C). Multivariate Cox regression analysis further identified 10 independent prognostic genes, and a risk model was constructed using these genes: riskscore = −0.1229087*CCL19 + 0.7138989*GABRA4 − 0.3249750*LINC01016 + 1.1752016 *LINC01234 + 1.2721024*MAFA + 0.2877169*SHCBP1 + 0.9271663*SLITRK3 + 0.2266221*SMR3B + 0.2268139*SPDYC + 0.1458826*SPINK8. Figure 3D illustrated the forestplot of included 10 genes. The TCGA-BRCA and GSE96058 cohorts’ subjects were dichotomized into low- and high-risk cohorts, based on the median value of their respective risk scores (Fig. 4A–F). K-M survival analysis was conducted on both cohorts, and the findings illustrated that BC patients in the low-risk group demonstrated markedly improved prognosis, relative to those in the high-risk group (Fig. 4G and I, *P* < .0001). Receiver operating characteristic analysis demonstrated that the AUCs of 1-, 3-, and 5-year survival in the TCGA-BRCA cohort were 0.805, 0.798, and 0.810, respectively, indicating the robustness of the predictive ability of the risk score model (Fig. 4H and J).

### 3.3. Clinical correlation analysis of the risk signature

**Figure 5.**
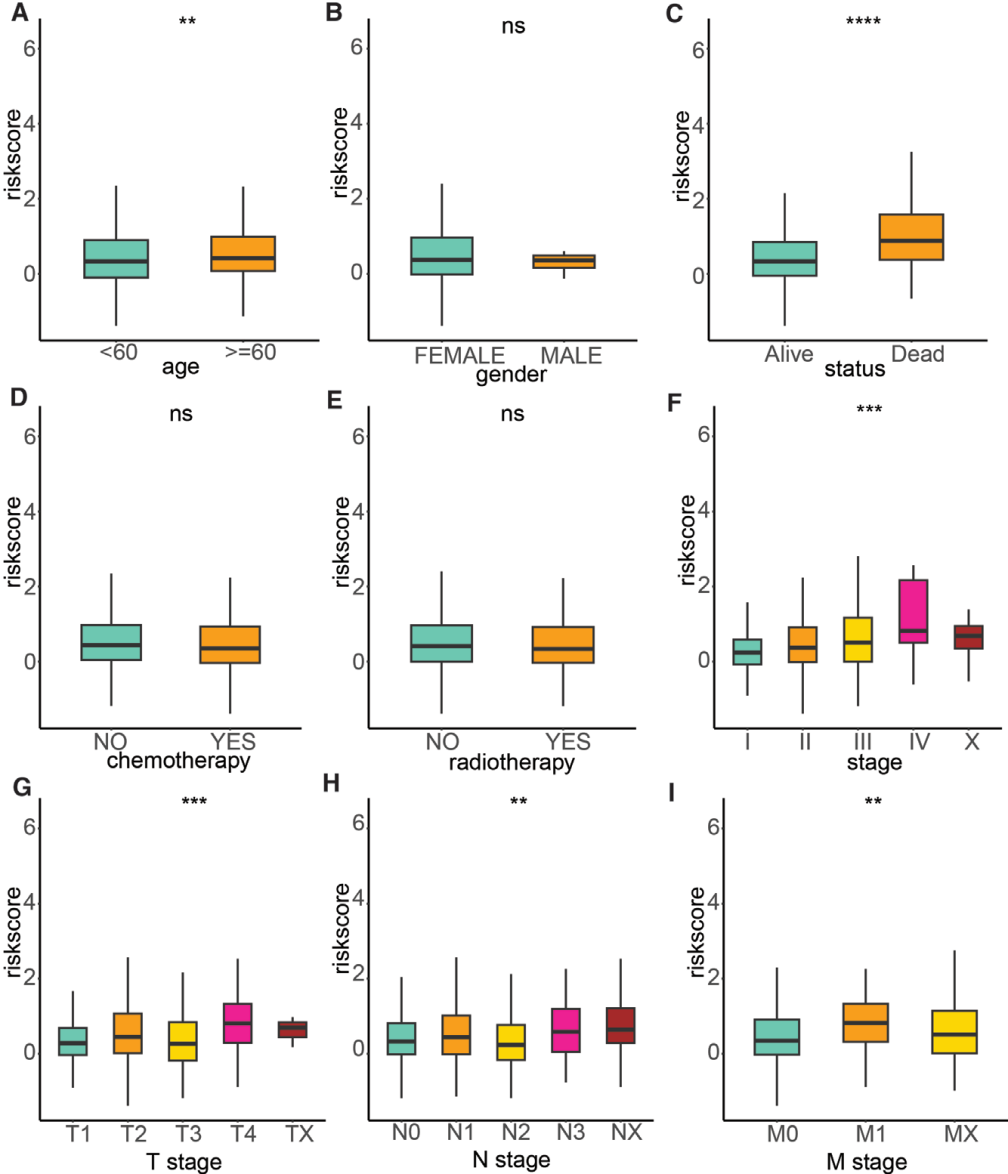
The association between the risk signature and clinical features. (A) Age; (B) Gender; (C) Status; (D) Chemotherapy; (E) Radiotherapy; (F) Stage; (G) T stage; (H) N stage; (I) M stage. *P < .05, **P < .01, ***P < .001, ****P < .0001.

To delve deeper into the clinical relevance of the signature, we examined its association with various clinical factors, including age, gender, survival status, chemotherapy, radiotherapy, stage, T stage, N stage, and M stage. Our analysis revealed that the risk score was significantly higher in patients aged 60 years and above compared to those younger than 60 years. Additionally, the risk score was found to be higher in deceased patients compared to their living counterparts. Furthermore, the risk score was significantly elevated in advanced stages of the disease relative to the early stages. Notably, advanced T, N, and M stages exhibited higher risk scores compared to their early stage counterparts (Fig. 5).

### 3.4. Immune infiltration and immunotherapy response

**Figure 6.**
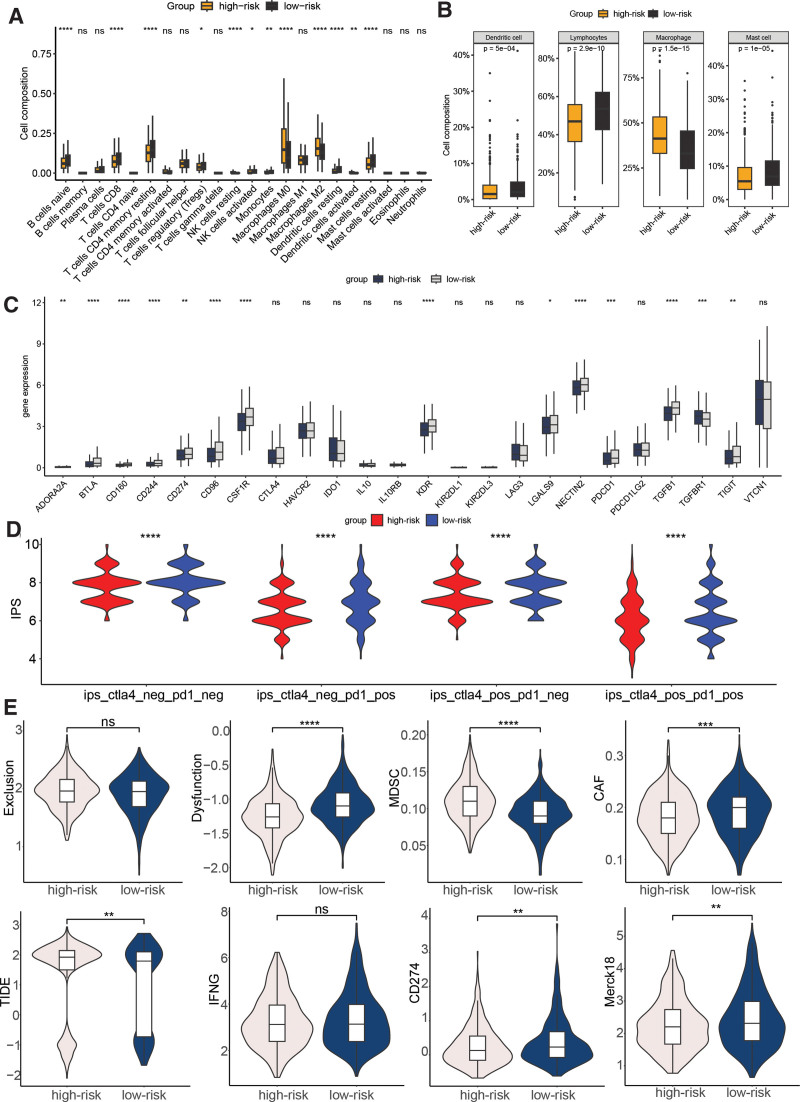
Relationship between BC risk model based on cell aging-related genes and immune infiltration and immune therapy response. (A) Infiltration analysis of 22 immune cell types based on the CIBERSORT algorithm. (B) Comparison results after categorizing the 22 immune cell types into 4 cell types. (C) Boxplot showing the expression difference of immune checkpoints in low- and high-risk THCA. (D) IPS score of the low- and high-risk THCA in the CTLA4-PD1-, CTLA4-PD1+, CTLA + PD1-, and CTLA + PD1 + subgroups. (E) Difference of the TIDE, Exclusion, Dysfunction, CD274, IFNG, Responder, Merck18, and MDSC score between low- and high-risk group. *P < .05, **P <= .01, ***P < .001, ****P < .0001. BC = breast cancer, IFNG = interferon-gamma, IPS = immunophenoscore, MDSC = myeloid-derived suppressor cells, TIDE = tumor immune dysfunction and exclusion.

Significant differences in immune infiltration was observed between the high- and low-risk groups, thereby shedding light on the clinical correlation of the signature. Specifically, the high-risk group demonstrated a significant enrichment of naive B cells, CD8 T cells, resting memory CD4 T cells, regulatory T cells, activated NK cells, monocytes, resting DCs, and resting mast cells. In contrast, the low-risk group showed significant enrichment of resting NK cells, M0 and M2 macrophages, and activated DCs (Fig. 6A). The classification comparison of immune cell populations further emphasized the significant enrichments of DCs, lymphocytes, and mast cells in the high-risk group, while macrophages were significantly enriched in the low-risk group (Fig. 6B). In terms of immune checkpoints, ADORA2A, BTLA, CD160, CD244, CD274, CD96, CSF1R, KDR, LGALS9, NECTIN2, PDCD1, TGFB1, TGFBR1, and TIGIT displayed a significant disparity between the low- and high-risk cohorts (Fig. 6C, *P* < .01). The IPS was compared between the 2 groups, and the results demonstrated that the low-risk cohort presented significantly elevated IPS values, suggesting a more promising immunotherapeutic response potential (Fig. 6D). Furthermore, the TIDE score was lower in the low-risk group compared to the high-risk group, indicating a potential favorable response to immunotherapy. Additionally, the low-risk group presented with higher T cell dysfunction, CD274, Merck18, and cancer-associated fibroblast levels, and lower myeloid-derived suppressor cells level (Fig. 6E).

### 3.5. GO and KEGG pathways associated with the risk signature

**Figure 7.**
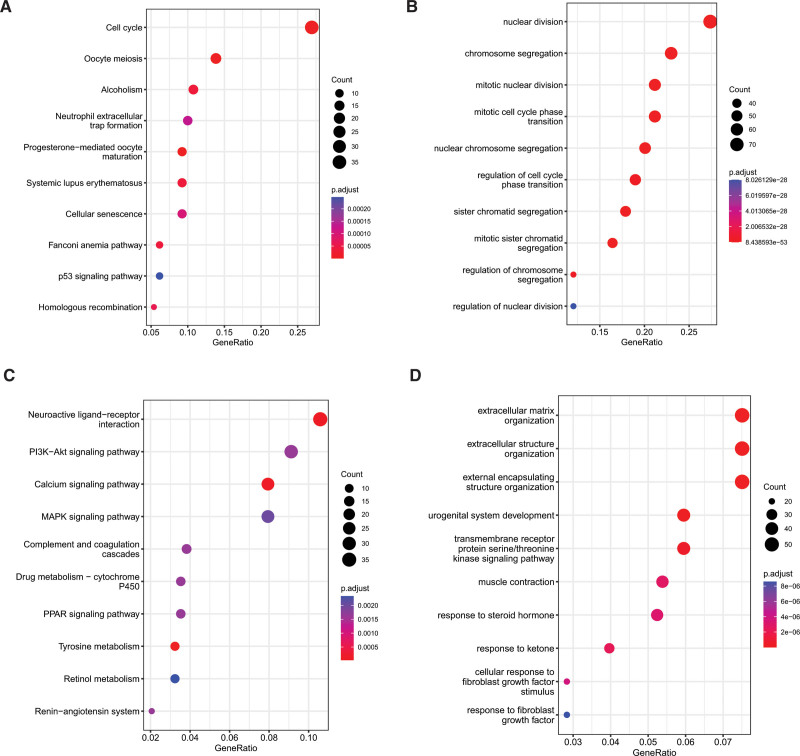
Functional enrichment analysis of low- and high-risk group. (A and B) KEGG pathway and GO enrichment results of upregulated genes. (C and D) KEGG pathway and GO enrichment results of downregulated genes. GO = gene ontology, KEGG = Kyoto encyclopedia of genes and genomes.

To scrutinize the plausible cellular functions and pathways related to the low- and high-risk cohorts, we conducted functional enrichment analysis on the DEGs between the 2 groups. The KEGG enrichment analysis of the upregulated genes highlighted that the 3 most enriched terms were cell cycle, oocyte meiosis, and alcoholism, as depicted in Figure 7A. Additionally, GO enrichment analysis of the upregulated genes revealed that the top 3 enriched terms were nuclear division, chromosome segregation, and mitotic nuclear division, as shown in Figure 7B. Regarding the downregulated genes, KEGG pathway enrichment analysis showed that the top 3 pathways were neuroactive ligand-receptor interaction, PI3K-AKT signaling pathway, and calcium signaling pathway (Fig. 7C), while the top 3 enriched GO terms were extracellular matrix organization, extracellular structure organization, and external encapsulating structure organization (Fig. 7D).

### 3.6. The CSRGs-derived risk signature was associated with drug sensitivity

**Figure 8.**
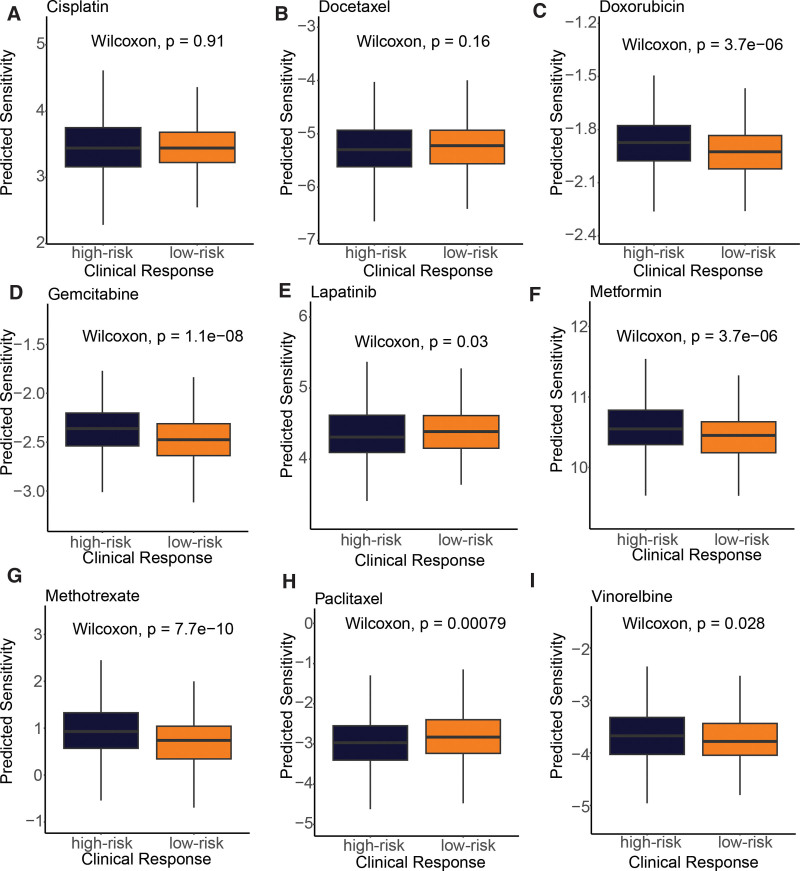
Drug sensitivity in low- and high-risk groups, including (A) cisplatin, (B) docetaxel, (C) docorubicin, (D) gemcitabine, (E) lapatinib, (F) metformin, (G) methotrexate, (H) paclitaxel, (I) vinorelbine.

To investigate the relationship between the cell senescence-based prognostic risk model and drug sensitivity, we performed a drug sensitivity analysis (Fig. 8) of 9 frequently adopted drugs in the TCGA-BRCA cohort for each patient. The study results indicated that patients in the high-risk group exhibited a more pronounced response to doxorubicin (*P* = 3.7e-06), gemcitabine (*P* = 1.1e-08), metformin (*P* = 3.7e-06), methotrexate (*P* = 7.7e-10), and vinorelbine (*P* = .028). Conversely, those in the low-risk group displayed a greater response to lapatinib (*P* = .03) and paclitaxel (*P* = .00079). These findings propose that the risk model holds the potential to predict chemotherapy response and elucidate the probable involvement of related genes in chemotherapy response.

### 3.7. Construction of a nomogram

**Figure 9.**
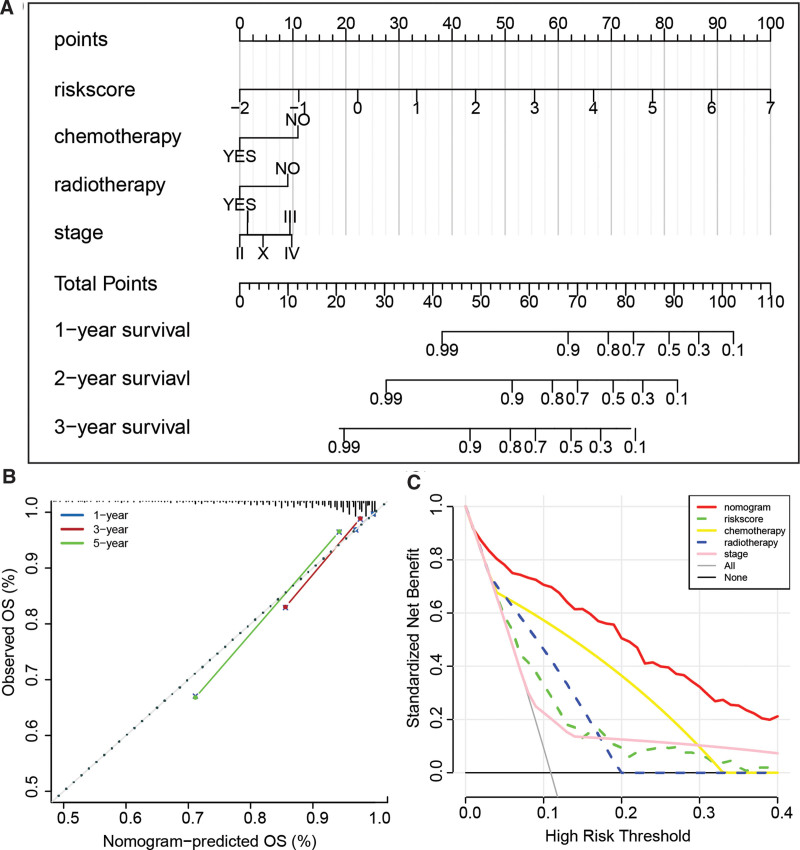
Construction and performance evaluation of a nomogram model based on cell aging-related genes derived BC risk score. (A) Nomogram model for the entire riskscore, chemotherapy, radiotherapy, and stage. (B) Calibration curve of the nomogram model in the TCGA-BRCA cohort. (C) Decision curve analysis comparing the performance of the nomogram model, riskscore, chemotherapy, radiotherapy, and stage in prognosis prediction. BC = breast cancer.

The outcomes of the univariate Cox regression analysis demonstrated that the prognosis of BC was significantly linked with various factors, such as risk score, age, chemotherapy, radiotherapy, and stage. Subsequently, the risk score, chemotherapy, radiotherapy, and stage were identified as independent risk factors for BC prognosis by multivariate Cox regression analysis, as illustrated in Table 1. To predict OS in BC patients, we created a nomogram incorporating these 4 factors, as demonstrated in Figure 9A. The nomogram achieved a C-index of 0.844 in the TCGA-BRCA cohort, indicating good predictive performance. Calibration curves in Figure 9B also confirmed the high predictive accuracy of our model. Moreover, decision curve analysis showed that our prediction model outperformed alternative strategies, as depicted in Figure 9C. In summary, our developed nomogram serves as a valuable instrument for predicting the prognosis of BC patients.

**Table 1:** Independent risk factors of BC identified by univariate and multivariate cox regression analysis.

Risk factors	Univariate cox				Multivariate cox			
	*P*	HR	95%CI		*P*	HR	95%CI	
Riskscore	0	2.719	2.262	3.267	0	2.626	2.148	3.21
Gender	.579	20.522	0	887157.68	.965	0	0	4.31E + 177
Age	.027	1.575	1.054	2.354	.569	1.14	0.726	1.789
Chemotherapy	0	0.22	0.137	0.354	.002	0.36	0.186	0.696
Radiotherapy	0	0.26	0.149	0.456	.01	0.354	0.16	0.779
Stage (I as reference)	0				0			
Stage II	.393	1.322	0.696	2.512	.533	1.375	0.505	3.745
Stage III	.006	2.603	1.317	5.142	.002	5.552	1.856	16.602
Stage IV	0	6.744	2.836	16.036	.007	5.857	1.619	21.188
Stage X	.028	3.021	1.127	8.101	.071	2.739	0.918	8.174
T stage (T1 as reference)	.293				.254			
T2	.641	1.124	0.688	1.835	.613	0.774	0.287	2.086
T3	.577	1.203	0.629	2.298	.58	0.682	0.176	2.649
T4	.04	2.334	1.039	5.246	.249	0.268	0.028	2.52
TX	.574	0.562	0.075	4.189	.023	0.026	0.001	0.598
N stage (N0 as reference)	.664				.204			
N1	.906	1.029	0.643	1.645	.54	0.808	0.408	1.598
N2	.28	1.402	0.759	2.591	.257	0.471	0.129	1.728
N3	.993	1.004	0.394	2.557	.757	0.782	0.165	3.711
NX	.247	1.677	0.699	4.025	.193	4.25	0.482	37.51
M stage (M0 as reference)	.149				.472			
M1	.059	2.408	0.969	5.987	.487	0.564	0.112	2.831
MX	.498	1.201	0.708	2.037	.468	1.272	0.664	2.435

BC = breast cancer.

## 4. Discussion

Cellular senescence is a complex and heterogeneous process that plays a critical role in maintaining tissue homeostasis and protecting cells against stress-induced damage. However, its precise contribution to mammary gland development and BC remains elusive. Senescence exerts regulatory effects on various cellular activities, and can have both beneficial and detrimental impacts on tumorigenesis. It may promote chemotherapy resistance and cancer recurrence, while also suppressing tumor growth by inhibiting senescence signals.^[10]^ Moreover, senescence is closely associated with tumor immunity. For instance, studies have shown that TSLP-activated CD4 + T cells can induce IFN-γ and TNF-α to drive BC cells into a senescent phenotype, which may facilitate evasion of tumor cells from the human immune system.^[11]^ Additionally, cytokines such as IL-6 and IL-8 can enhance the tumorigenic potential of the MCF-7 BC cell line by inducing a senescence/inflammation milieu that is self- and cross-enhanced.^[12]^ To provide a comprehensive understanding of BC senescence, a novel gene signature based on senescence-associated genes was developed, which can accurately predict prognosis and response to immunotherapy.

The gene signature identified in this study consists of ten genes: C-C motif chemokine ligand 19 (CCL19), GABRA4, LINC01016, LINC01234, MAFA, SHCBP1, SLITRK3, SMR3B, SPDYC, and SPINK8. CCL19 is a well-known mediator of homeostatic and physiological functions in primary and secondary lymphoid organs.^[13]^ However, recent research has suggested that CCL19 might also serve as a potential prognostic biomarker and regulator of the tumor immune microenvironment in BC.^[14]^ LINC01016 exhibits elevated expression levels in BC and has been found to have prognostic significance with respect to BC survival.^[15]^ Furthermore, LINC01016 has been reported to promote the malignant phenotype in endometrial cancer cells by acting as miRNA sponge.^[16]^ LINC01234, another novel lncRNA, has been implicated in various cancers, where it exerts a proto-oncogenic effect by modulating cellular processes such as cell cycle progression, migration, and apoptosis.^[17–19]^ SHC binding and spindle-associated 1 (SHCBP1) is a protein that specifically binds to the SH2 domain of Src homology-collagen, and its abnormal expression has been linked to cancer in various systems.^[20]^ SLITRK3, a homologous transmembrane protein, has been implicated in the risk rating and prognosis of gastrointestinal stromal tumors.^[21]^ Moreover, increased expression of SLITRK3 in LUSC has been linked to unfavorable clinical outcomes, potentially attributable to its function in facilitating SLITRK3-mediated activation of NTRK3, thereby promoting a cancer stem cell phenotype.^[22]^ Despite the identification and study of some genes, those involved in BC remain largely unexplored. Therefore, further research is crucial.

The microenvironment of BC comprises multiple cell types, such as fibroblasts, DCs, macrophages, and lymphocytes.^[23]^ This study demonstrated that the levels of immune cell infiltration differed significantly among different risk groups, with the low-risk group having higher levels of DCs, lymphocytes, and mast cells, and lower levels of macrophages. DCs play a critical role in inducing anti-tumor responses by cross-presenting antigens to CD4 + and CD8 + T cells, thereby activating them to attack neoplastic cells.^[24]^ The presence of immature DCs in the tumor-associated stroma impairs their ability to stimulate antitumor immunity, and these immature DCs produce angiogenic factors that promote endothelial cell migration and actively facilitate tumor growth.^[25]^ However, DC maturation suppresses this angiogenic trait.^[25]^ Conversely, mature DCs implanted at the primary tumor site can contribute to reduced metastasis and improved clinical outcomes.^[26]^ Tumor-associated macrophages promote BC growth, angiogenesis, and metastasis by producing pro-tumor factors, including VEGF.^[27]^ High TAM levels are associated with poorer prognosis, and TAM exhaustion or reprogramming may be a potential therapeutic strategy.^[27,28]^ Tumor-infiltrating lymphocytes also play an important role in the tumor microenvironment, and our analysis showed that low-risk patients had higher lymphocyte infiltration, mainly represented by naive B cells, CD8 + T cells, resting memory CD4 + cells, Tregs, and activated NK cells. T regulatory cells generally function to prevent autoimmune disorders by inhibiting self-reactive T cells. However, within the tumor microenvironment, they suppress anti-tumor responses.^[29]^ Furthermore, the infiltration rate of CD8 + effector T cells has prognostic value for the survival of patients with BC and is independent of traditional clinical factors, such as tumor grading and lymph node staging.^[30]^

Analysis of functional enrichment suggests that several pathways, such as the cell cycle, neutrophil extracellular trap (NET) formation, neuroactive ligand-receptor interaction, PI3K-AKT signaling pathway, and calcium signaling pathway, may play a role in both low- and high-risk BC. NETs are structures that are formed by neutrophils as a defense mechanism against invading pathogens. In addition, regulatory roles in tumor metastasis have also been attributed to neutrophils, as research indicates that cancer cells can stimulate the process of NETosis, resulting in the formation of NET that facilitate tumor growth and metastasis.^[31,32]^ Research studies have indicated that Cathepsin C plays a crucial role in the regulation of neutrophil infiltration and NET formation, promoting lung metastasis of BC.^[33]^ Furthermore, the phosphatidylinositol 3-kinase (PI3K)/AKT pathway is known to play significant roles in multiple biological and cellular processes, and has been implicated in the pathogenesis of various types of cancers.^[34,35]^ Altered calcium signaling pathways, resulting from mutations or changes in expression of calcium channels and effectors, can impact all facets of cancer in solid tumors by disrupting the transport of calcium ions (Ca2+) in the endoplasmic reticulum or mitochondria, ultimately affecting cellular apoptosis.^[36]^

## 5. Conclusion

To summarize, we developed molecular clusters for BC based on CSRGs, which exhibited significant differences in both prognosis and immune infiltration. Differential expression genes were identified between the different clusters, and 10 independent prognostic genes were screened to construct a BC prognosis evaluation model with strong predictive ability for OS. Our findings indicated that older age and later clinical stages corresponded to lower risk, and patients in different risk groups showed substantial differences in immune cell infiltration, drug sensitivity, and response to immune therapy, suggesting that the model may have utility in predicting these outcomes. Taken together, our novel senescence-related model may hold implications for the development of targeted therapies in the context of BC.

## Authors contributions

**Conceptualization:** Tao Pan, Zhengfang Hu.

**Data curation:** Tao Pan, Zhengfang Hu, Suzhan Zhang.

**Formal analysis:** Tao Pan, Dongyan Xu, Yiding Chen.

**Funding acquisition:** Tao Pan.

**Investigation:** Zhengfang Hu.

**Methodology:** Dongyan Xu.

**Project administration:** Dongyan Xu.

**Resources:** Dongyan Xu.

**Software:** Tao Pan, Yunxiang Zhou.

**Supervision:** Dongyan Xu.

**Validation:** Zhengfang Hu, Yiding Chen.

**Visualization:** Tao Pan, Dongyan Xu.

**Writing – original draft:** Tao Pan, Zhengfang Hu.

**Writing – review editing:** Tao Pan.
